# Peer Review of “Risk Factors of SARS-CoV-2 Infection: Global Epidemiological Study”

**DOI:** 10.2196/31926

**Published:** 2021-08-26

*This is a peer-review report submitted for the paper "Risk Factors of SARS-CoV-2 Infection: Global Epidemiological Study".*

## Round 1 Review

Thank you for your submission [1]. I don’t have any suggestions except that a lot has changed since late winter 2020; are you planning to update this manuscript to represent the most recent data? Or alternatively, it may be a good idea to look into the most recent picture and compare it with the findings of the present manuscript.
